# Hypoxia-induced MIF induces dysregulation of lipid metabolism in Hep2 laryngocarcinoma through the IL-6/JAK-STAT pathway

**DOI:** 10.1186/s12944-022-01693-z

**Published:** 2022-08-30

**Authors:** Minlan Yang, Sa Wu, Weisong Cai, Xiaoping Ming, Yuhao Zhou, Xiong Chen

**Affiliations:** Department of Otorhinolaryngology, Head and Neck Surgery, Zhongnan Hospital of Wuhan University, Wuhan, China

**Keywords:** Hypoxia, MIF, Lipid metabolism, IL-6/JAK-STAT pathway

## Abstract

**Purpose:**

Hypoxia is a common feature of laryngocarcinoma. Alterations in lipid metabolism are an important metabolic rewiring phenomenon for malignant cells to maintain their rapid proliferation in the hypoxic microenvironment, which makes most cancers, including laryngocarcinoma, difficult to cure. However, the mechanisms involved in lipid metabolism in laryngocarcinoma is still unclear. This study aimed to clarify the changes in lipid metabolism of laryngocarcinoma cells under hypoxic conditions and explore the related mechanisms.

**Methods:**

Hep2 cells were incubated in a normoxic or hypoxic environment (5% CO_2_ and 1% O_2_) at 37 °C for 24 h. CCK-8 cell viability assay and colony formation assay were performed to detect cells proliferation. And lipid metabolic indices including TG and NEFA were determined by kits. The mechanism involved in the regulation of lipid metabolism was explored by RNA-seq and bioinformatic analysis. The MIF inhibitor ISO-1 and JAK inhibitor XL019 were used to verify the mechanism. Finally, a tumour xenograft model was applied to further verify these results in vivo.

**Results:**

Hypoxia promoted cell proliferation and increased the levels of TG and NEFA in Hep2 cells. Three genes, MIF, ENO2, and LDHA, that were screened by the intersection of hypoxia gene sets and fatty gene sets and were verified by qPCR. The MIF levels were elevated when cells were exposed to hypoxia. Through GSEA and RNA-seq analysis, the JAK/STAT pathway was screened. Hypoxia increased MIF levels and activated the IL-6/JAK/STAT pathway. The MIF inhibitor ISO-1inhibited cell proliferation under hypoxia and reversed the change in TG levels and IL-6 levels. And ISO-1 reversed the expression pattern of the screened genes in the JAK/STAT pathway. Finally, a tumour xenograft model further verified these results in vivo.

**Conclusion:**

Hypoxia induced reprogramming of lipid metabolism in laryngocarcinoma cells through the MIF/IL-6/JAK-STAT pathway. This study revealed one mechanism that allows laryngocarcinoma cells to adapt to the hypoxic tumour microenvironment. Therefore, a drug targeting the MIF/IL-6/JAK-STAT pathway might be a promising therapeutic option for the treatment of laryngocarcinoma.

**Supplementary Information:**

The online version contains supplementary material available at 10.1186/s12944-022-01693-z.

## Introduction

Hypoxia is a general phenomenon in the tumour microenvironment(TME). In the process of tumour progression, there is a rapid proliferation of tumour cells. Thus, the vascular network cannot be established quickly, and the new blood vessels are structurally abnormal. This results in a decrease in the oxygen content in the microenvironment, a lack of nutrients and the accumulation of acidic substances. In the hypoxic TME, tumour cells can improve their adaptability by changing their metabolic pathways and inhibiting the antitumor effects of immune cells, making them prone to invasion, metastasis, and drug resistance. Therefore, the hypoxic TME increases the difficulty of tumour treatment. Proteins, lipids and nucleic acids are important components of the biological membranes and structural units of cells. Lipids are used for metabolism and energy storage and play vital roles in the molecular signaling of a variety of cellular activities. The regulation of lipid metabolism, such as lipid uptake, hydrolysis and synthesis, is essential for maintaining cell homeostasis. In the process of tumour progression, the availability of nutrients in the TME is constantly changing, and tumour cells alter lipid metabolism to maintain survival, malignant proliferation, migration, invasion, and metastasis. To adapt to the hypoxic TME, the metabolism of tumour cells will also undergo corresponding changes. The hypoxic microenvironment enhances lipogenesis through the HIF-dependent regulation of proteins involved in fatty acid(FA) uptake, hydrolysis, synthesis, storage and usage, enhancing cancer progression and hypoxia-induced chemoresistance [1–3]. Hypoxia-induced alterations to lipid metabolism promote malignancy progression and have been explored in various cancers. In prostate cancer, hypoxic cells accumulate more lipids, and FA oxidation is decreased. These responses protect cancer cells from endoplasmic reticulum stress and oxidative and play important roles in accelerating cell proliferation [4]. In clear cell renal cell carcinoma(ccRCC), HIF1 and HIF2 repressed the target gene CPT1A. This repression thus reduces FA transport into the mitochondria and redirects fatty acids to lipid droplets for storage, which is essential for ccRCC tumorigenesis [5]. In the hypoxic TME, HIF-2α overexpression promotes steatotic hepatocellular carcinoma progression by activating lipid synthesis [6]. Inhibition of lipid storage decreased the survival of cells exposed to hypoxia-reoxygenation and strongly inhibited tumorigenesis of multiple cancers [7]. Laryngeal cancer is a common disease in otorhinolaryngology, and its incidence rate has increased in recent years. This study aimed to explore the effects of hypoxia on lipid metabolism in laryngocarcinoma cells.

## Methods and materials

### Cell culture

Hep2, the human laryngocarcinoma cell line, was obtained from the Medical Science Research Center, Zhongnan Hospital of Wuhan University, and maintained in DMEM/HIGH GLUCOSE supplemented with 10% fetal bovine serum, plus the 1X penicillin–streptomycin solution (Bioshrp, Hefei, China). Cells were cultured in a humidified incubator at 37℃, and 5% CO_2_. 1% O_2_ and 5% CO_2_ gas mixture was chosen for hypoxia experiment. MIF inhibitor ISO-1 (SML0472) was purchased from Sigma-Aldrich(Sigma-Aldrich, Shanghai, China), and JAK inhibitor XL019 was purchased from Beyotime Biotechnology (Beyotime, Shanghai, China). Drug concentrations were 25 µM(ISO-1) and 100 nM(XL019).

### Western blotting

Cell samples were lysed with RIPA buffer protease inhibitor cocktail (Beyotime, Shanghai, China). Protein was separated by 10% SDS-PAGE, transferred onto 0.45 μm PVDF membranes, blocked and immunoprobed. Finally, chemiluminescence kit (Beyotime, Shanghai, China) was applied to detect the signals.

### Quantitative real-time PCR

TRIzol reagent (Invitrogen, Carlsbad, CA) was used to extract total RNA, and cDNA synthesis was performed following the protocol of Transcriptase Kit (Thermo, Waltham, MA). And qRT-PCR was conducted on a CFX96 Connect (Bio-Rad, Hercules, CA) using a SYBR Green PCR kit (Vazyme Biotech, Nanjing, China). The mRNA expression levels were calculated by using the 2^−ΔΔCt^ method. Results are shown as fold-change relative to the normal group or the DMSO group. Primer sequences were listed in Supplementary Data.

### CCK-8 cell viability assay

Hep2 cells treated with hypoxia and ISO-1 and control cells were seeded into 96-well plates at 2000 cells per well. Cell viability was evaluated by replacing with fresh medium containing 10% Cell Counting Kit-8 (CCK8) reagent (Biosharp, Hefei, China) with incubation at 37℃for 2 h and plates were measured at 450 nm by a microplate reader (Thermo Fisher, Waltham, MA). Each group was measured every 24 h for 7 consecutive days.

### Colony formation assay

Hep2 cells treated with hypoxia and ISO-1 and control cells were seeded at 500 cells into 6-well plates per well. The cell colonies were fixed and stained with 0.3% crystal violet in ethanol for 15 min until the colonies were obviously visible to the naked eye. Finally, pictures of the whole well were taken.

### ELISA assay

MIF and IL-6 concentrations were tested by ELISA kits based on the manufacturer’s protocol. Quantitative IL-6 ELISA assay kit was purchased from QuantiCyto (QuantiCyto, Shenzhen, China), Quantitative MIF ELISA assay kit was purchased from Elabscience (Thermo, Waltham, USA), and TG, NEFA levels were assayed by assay kits (Nanjing Jiancheng Bioengineering Institute, Nanjing, China).

### Bioinformatics analysis

Hypoxia gene sets and fatty gene sets were downloaded from Gene Set Enrichment Analysis(GSEA)( http://www.gsea-msigdb.org/gsea/index.jsp) and Venn diagrams were used to analyze the intersection of the two gene sets. Gene expression profiles of larynx were downloaded from TCGA (https://portal.gdc.cancer.gov/). GSEA was performed using GSEA software 4.0.3(UC San Diego and Broad Institute, La Jolla, CA).

### RNA-seq

Hep2 cells maintained for 24 h in normoxia as the control group and cultured for 24 h in hypoxia as the treatment group were used for transcriptome sequencing at BGI (BGI, Shenzhen, China) (three independent replicates per group). The relative analysis was carried out on the website(https://biosys.bgi.com).

### Immunohistochemistry staining

Xenograft tumour tissues were fixed, and dehydrated using gradient ethanol. Then, IHC staining was conducted following the manufacturer's instructions for the IHC kit(Maixin, Fuzhou, China). Anti-Ki-67 antibodies (dilution, 1:500; cat. no. ab15580; Abcam, Cambridge, UK) were incubated overnight at 4˚C. Finally, Image J v1.8.0 (National Institutes of Health, Bethesda, MA)was used for the analysis.

### Animal experiment

All mice were maintained at Zhongnan Hospital of Wuhan University and all animal experiments were conducted in accordance with Zhongnan Hospital animal ethics committee(NO. ZN2021049, June 8, 2021). Male BABL/c nude mice (4 weeks old) were purchased from Gempharmatech (Nanjing, China). Hep2 cells were collected by trypsinization and washed three times with pre-chilled PBS. Then cells were resuspended at 2 × 10^7^ cells/mL using pre-chilled saline, and each mouse was subcutaneously injected 200 μL into the right anterior flank. After 8 days, the mice were randomly divided into the treatment group and the control group(5 mice per group). The treatment group was treated with ISO-1(2.5 mg/kg, intraperitoneally, every day) and the control group was treated with saline(equal volume per weight, intraperitoneally, every day). Tumour volume was measured using a caliper every other day. Finally, mice were sacrificed, and tumours were removed, photographed, weighed and collected for ELISA and immunohistochemistry.

### Statistical analysis

All cellular experiments were repeated for at least three independent times. All data were presented as the mean ± SD. SPSS 20.0 software (IBM Corporation, Armonk, USA) was used for statistical analysis. T-test and ANOVA were used for continuous variables, and Chi-square test was used to analyze categorical variables. A difference of *P* < 0.05 was considered statistically significant.

## Results

### Hypoxia affects lipid metabolism in Hep2 cells

**Fig. 1:**
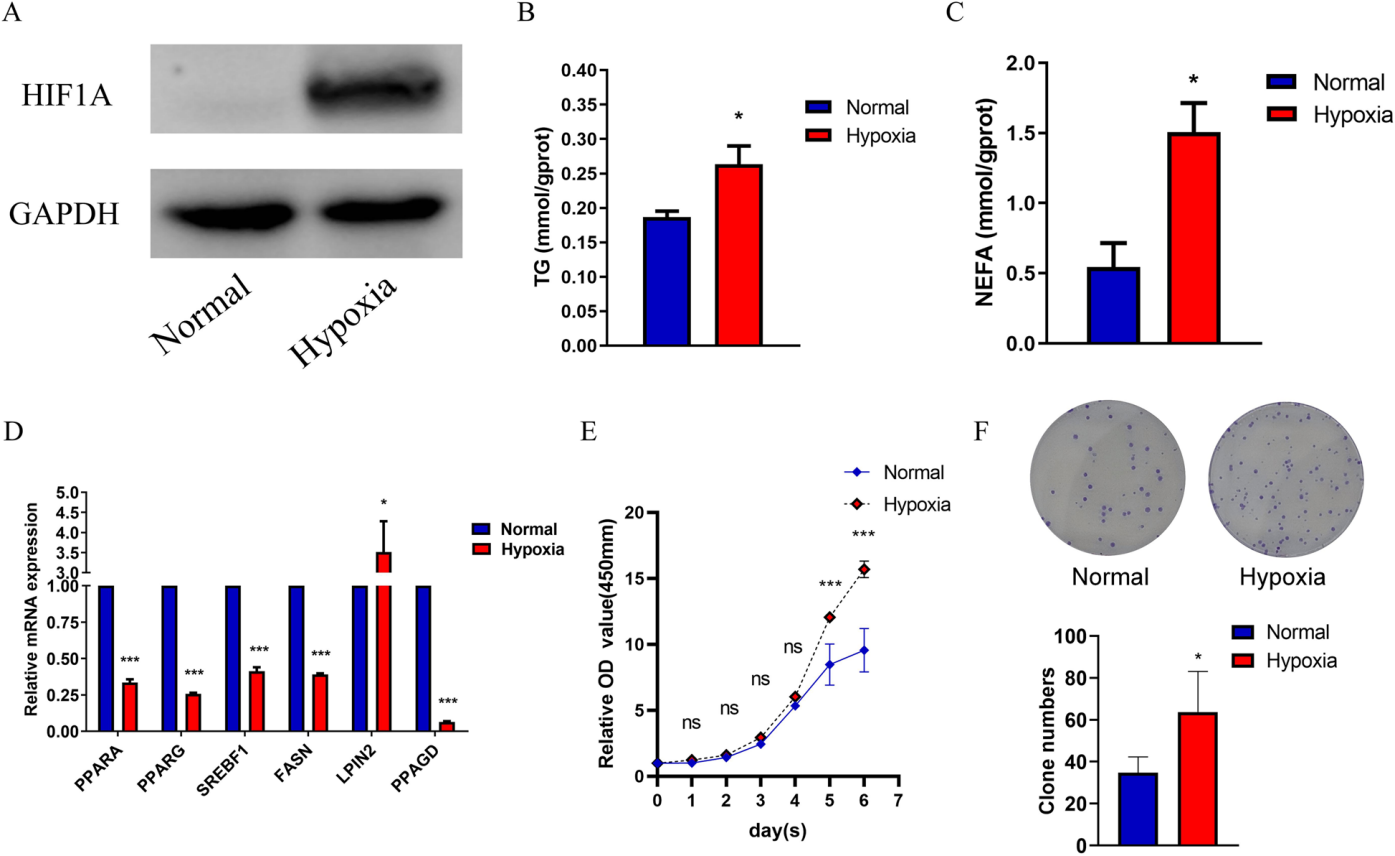
Hypoxia affects lipid metabolism in Hep2 cells. Hep2 cells were treated under a hypoxia environment for 24 h. **A** The protein level of HIF1A was assessed by western blot. **B** TG concentration was assessed by TG assay. **C** NEFA concentration was assessed by NEFA assay. **D** Expressions of lipid metabolism-related genes were screened out by qRT-PCR. **E** CCK8 assay assessed the proliferation of Hep2 cells incubated in normoxia and hypoxia. **F** Representative images and statistical analysis of colony formation assay. The data are presented as mean ± SD. *P < 0.05; ***P < 0.001

Hypoxia inducible factor 1 subunit alpha (HIF1A), which is an adaptive factor in the hypoxic environment, was found to be more highly expressed when Hep2 cells were exposed to a hypoxic environment of 1% O_2_ (Fig. 1A). To determine whether hypoxia affects lipid metabolism in laryngocarcinoma cells, the level of triglycerides (TGs), which are components of lipids, were detected. The TG level of Hep2 cells was markedly increased after exposure to hypoxia (Fig. 1B). In addition, nonesterified free fatty acids (NEFAs) are the major component of triglycerides, and we also found that hypoxia increased NEFA levels in Hep2 cells (Fig. 1C). Furthermore, we found that the expression pattern of some lipid metabolism-related genes was changed. The expression levels of PPARA, PPARG, SREBF1, FASN, and PPARD were downregulated, and LPIN2 was upregulated when exposed to hypoxia (Fig. 1D). The cell proliferation was evaluated by CCK-8 assay. As is shown, hypoxia promotes Hep2 cell proliferation (Fig. 1E). The result of colony formation assay also showed that clone numbers of the hypoxia group compared to the control group. These results suggest that hypoxia might play an important role in regulating lipid metabolism in laryngocarcinoma cells (Fig. 1F).

### MIF may be a key factor in the regulation of lipid metabolism by hypoxia

**Fig. 2:**
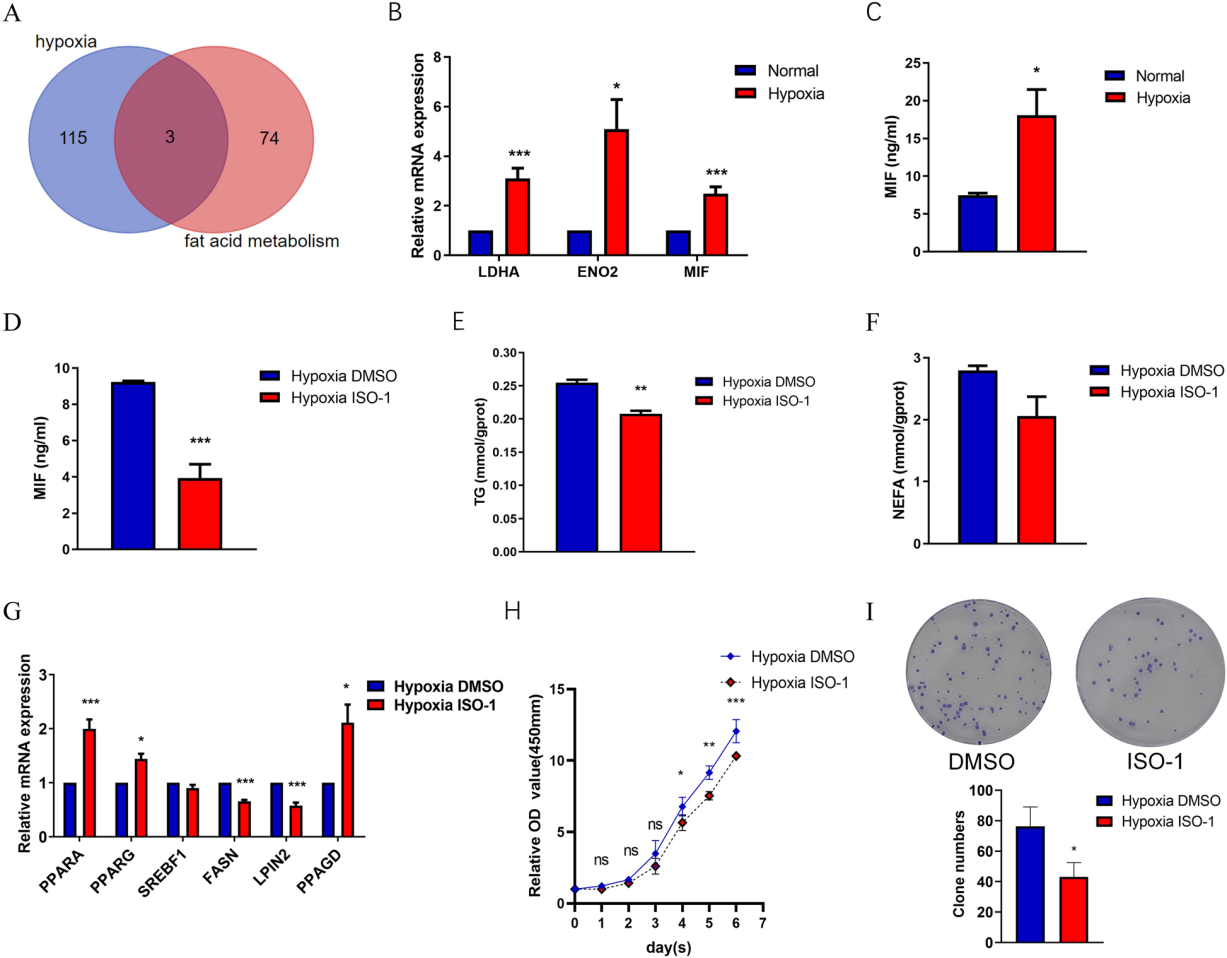
MIF may be a key factor in hypoxia regulating lipid metabolism. **A** Venn diagram showed the intersection of hypoxia gene sets and fatty gene sets. **B** The mRNA expression of MIF, ENO2 and LDHA were verified by qRT-PCR. **C** The protein level of MIF was assessed by ELISA assay. Hep2 cells were treated with 25 µ M ISO-1. Then MIF protein level (**C**), TG (**D**) and NEFA (**E**) concentration were assessed. **F** NEFA concentration was assessed by NEFA assay. **G** QRT-PCR verified the mRNA expression of lipid metabolism-related genes. **H** CCK8 assay assessed the proliferation of Hep2 cells. **I** Representative images and statistical analysis of colony formation assay. The data are presented as mean ± SD. *P < 0.05; **P < 0.01; ***P < 0.001

As hypoxia is an important feature of the solid tumour microenvironment, malignant cells confront the challenges of high growth rates and a limited supply of O_2_. Therefore, cancer cells change their pattern of metabolism to adapt to hyperproliferation. To explore whether lipid metabolism is involved when laryngeal cancer is exposed to a hypoxic microenvironment, we analyzed hypoxia gene sets and fatty gene sets. Three genes, lactate dehydrogenase A (LDHA), enolase 2 (ENO2) and macrophage migration inhibitory factor (MIF), may be involved in lipid metabolism in laryngeal cancer cells (Fig. 2A). Consistent with the intersection of the hypoxia gene sets and fatty gene sets that was determined by bioinformatics analysis, hypoxia significantly upregulated the expression of LDHA, ENO2 and MIF (Fig. 2B). As MIF is a classical proinflammatory cytokine that is secreted by immune cells and certain other cell types, the protein level of MIF was assessed. The protein level of MIF was increased when cells were exposed to hypoxia. Since MIF is also a key factor in lipid metabolism disturbance, we hypothesized that MIF signaling might be important for hypoxia-induced lipid metabolic disorders. To verify whether MIF is the main factor in hypoxia affecting lipid metabolism, ISO-1, an MIF antagonist, was used. An ISO-1 concentration of 25 μM significantly inhibited the protein level of MIF, and in the hypoxic environment (Fig. 2C, D), ISO-1 evidently decreased the TG level of Hep2 cells(Fig. 2E). Additionally, the NEFA level of Hep2 cells was slightly decreased, although there was no statistical difference (Fig. 2F). To further verify the role of MIF in lipid metabolism, MIF was then added, and the expression of lipid metabolism-related genes was reversed with ISO-1 treatment (Fig. 2G). Moreover, results of the CCK8 assay and the colony formation assay showed that ISO-1 inhibited cell proliferation and clone formation when Hep2 cells were exposed to hypoxia(Fig. 2H, I).

### JAK/STAT signaling is involved in MIF-regulating pathways in laryngeal cancer

**Fig. 3:**
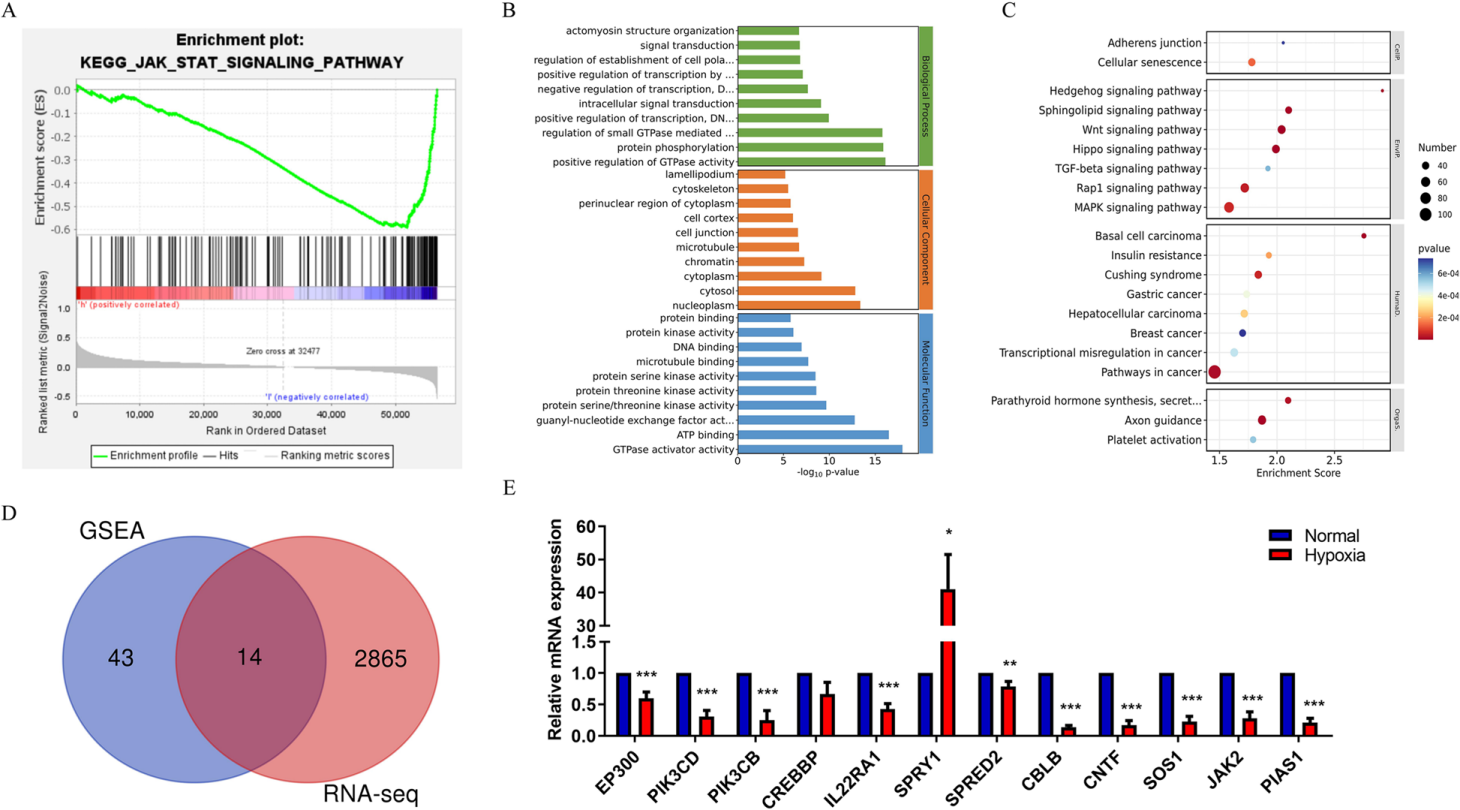
JAK/STAT signaling is involved in MIF regulating pathways in laryngeal cancer. **A** GSEA analysis showed JAK/STAT signaling is involved in MIF regulating pathways in laryngeal cancer. **B** GO analysis of differentially expressed genes. **C** KEGG analysis of differentially expressed genes. **D** Venn diagram showed the intersection of JAK/STAT gene sets and differently expressed genes in RNA seq. **E** qRT-PCR showed the intersection gene expression is basically consistent with the analysis under hypoxia. The data are presented as mean ± SD. *P < 0.05; **P < 0.01; ***P < 0.001

GSEA was used to screen the pathways involved in MIF-regulating pathways in laryngeal cancer (gene sets enriched in phenotype l (251 samples)). The top 10 pathways including the JAK STAT SIGNALLING PATHWAY were listed in Table 1. MIF is an upstream modulator of IL-6 and the IL-6/JAK/STAT pathway and has a vital impact on the growth and development of many human cancers. Therefore, we hypothesized that hypoxia-induced MIF regulates lipid metabolism by activating IL-6/JAK/STAT signaling (Fig. 3A). To investigate the possible effects of hypoxia on hep2 cells, RNA-seq was performed. And Fig. 3B and C showed the functional enrichment result of differentially expressed genes. Fourteen genes were found to be consistent by the intersection of JAK/STAT pathway gene sets and RNA-seq (Fig. 3D). RT-qPCR was used for verification, and the gene expression profile was consistent with the analysis (Fig. 3E). These results confirm that hypoxia activates the JAK/STAT signaling pathway.

**Table 1:** GSEA analysis of MIF in laryngeal carcinoma

Description	setSize	enrichScore	NES	*P* value
KEGG_JAK_STAT_SIGNALING_PATHWAY	155	-0.59	-2.19	0.003
KEGG_ADHERENS_JUNCTION	73	-0.65	-2.11	0.005
KEGG_ARRHYTHMOGENIC_RIGHT_VENTRICULAR_CARDIOMYOPATHY_ARVC	74	-0.65	-2.08	0.007
KEGG_ECM_RECEPTOR_INTERACTION	84	-0.71	-2.03	0.011
KEGG_HUNTINGTONS_DISEASE	180	0.68	2.31	0
KEGG_ALZHEIMERS_DISEASE	165	0.63	2.23	0
KEGG_OXIDATIVE_PHOSPHORYLATION	131	0.78	2.21	0
KEGG_PARKINSONS_DISEASE	128	0.75	2.21	0
KEGG_GLUTATHIONE_METABOLISM	49	0.68	2.11	0.003
KEGG_SPLICEOSOME	127	0.7	2.1	0.003

### Hypoxia induces MIF to regulate lipid metabolism by activating IL-6/JAK/STAT signaling.

**Fig. 4:**
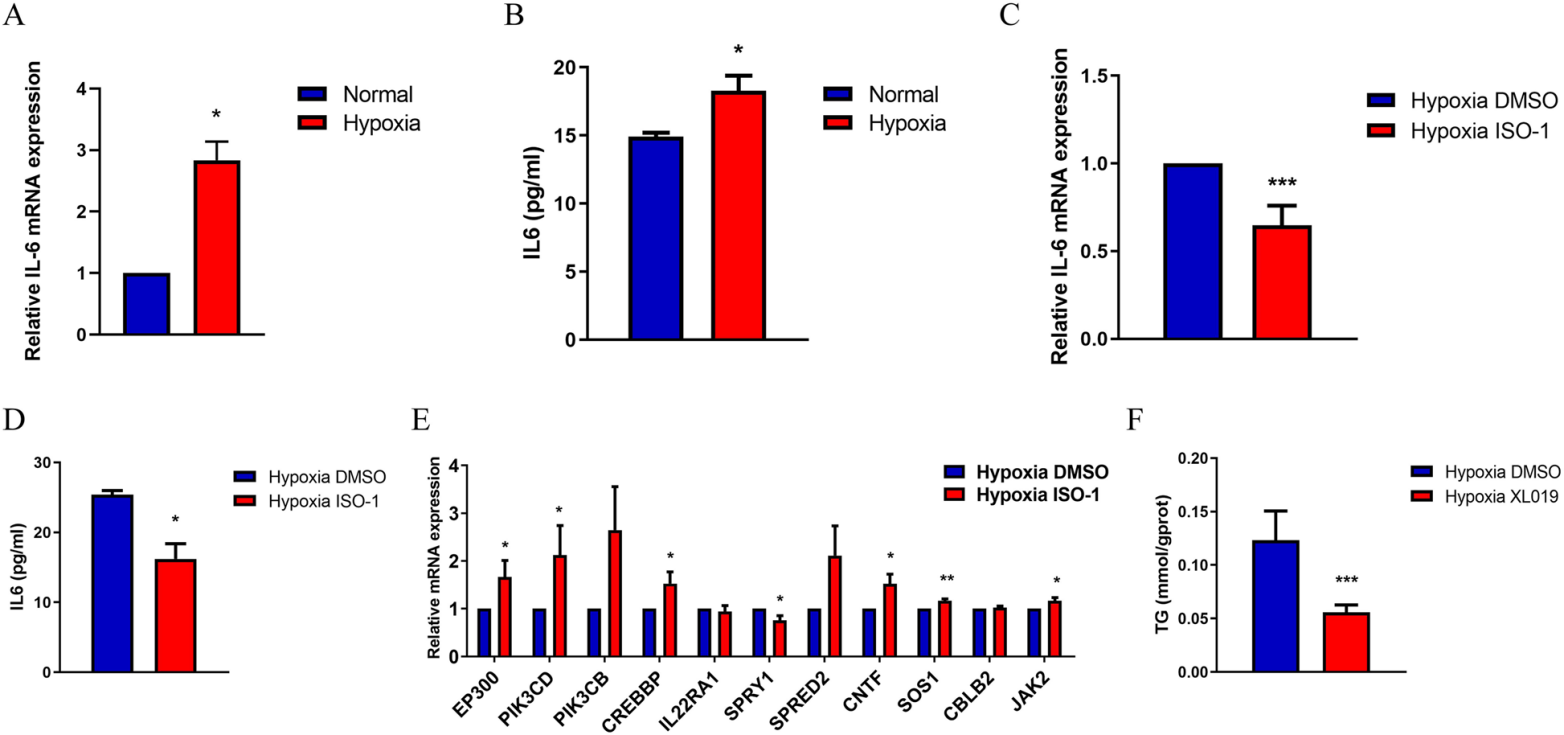
Hypoxia induces MIF regulating lipid metabolism through activating IL-6/JAK/STAT signaling. Hep2 cells were treated with 25 μM ISO-1 under hypoxia. Then the mRNA (**A**) and protein level (**B**) of IL-6 were assessed by qRT-PCR and western blot. **C** The intersection gene expressions of JAK/STAT gene sets and differently expressed genes in RNA seq were verified. **D** TG concentration of Hep2 cells treated with XL019 or solvent under hypoxia. The data are presented as mean ± SD. *P < 0.05; **P < 0.01; ***P < 0.001

When exposed to hypoxia, IL-6 was increased at the mRNA and protein levels (Fig. 4A, B). ISO-1 significantly reversed the hypoxia-induced upregulation of IL-6 (Fig. 4C, D). RT–qPCR assays were applied to test the mRNA expression of JAK/STAT pathway genes and revealed that ISO-1 reversed the expression pattern of the JAK/STAT pathway gene profile. These results suggest that hypoxia activates the IL-6/JAK/STAT pathway via MIF (Fig. 4E). To further verify that MIF regulates lipid metabolism by activating the IL-6/JAK/STAT pathway, the JAK inhibitor XL019 was used, and the TG level of Hep2 cells was significantly decreased with XL019 treatment when cells were exposed to hypoxia (Fig. 4F). These results confirm that the inhibition of JAK signaling reversed hypoxia-induced abnormalities in lipid metabolism.

### The MIF antagonist ISO-1 inhibits the tumorigenicity of Hep2 cells in vivo.

**Fig. 5:**
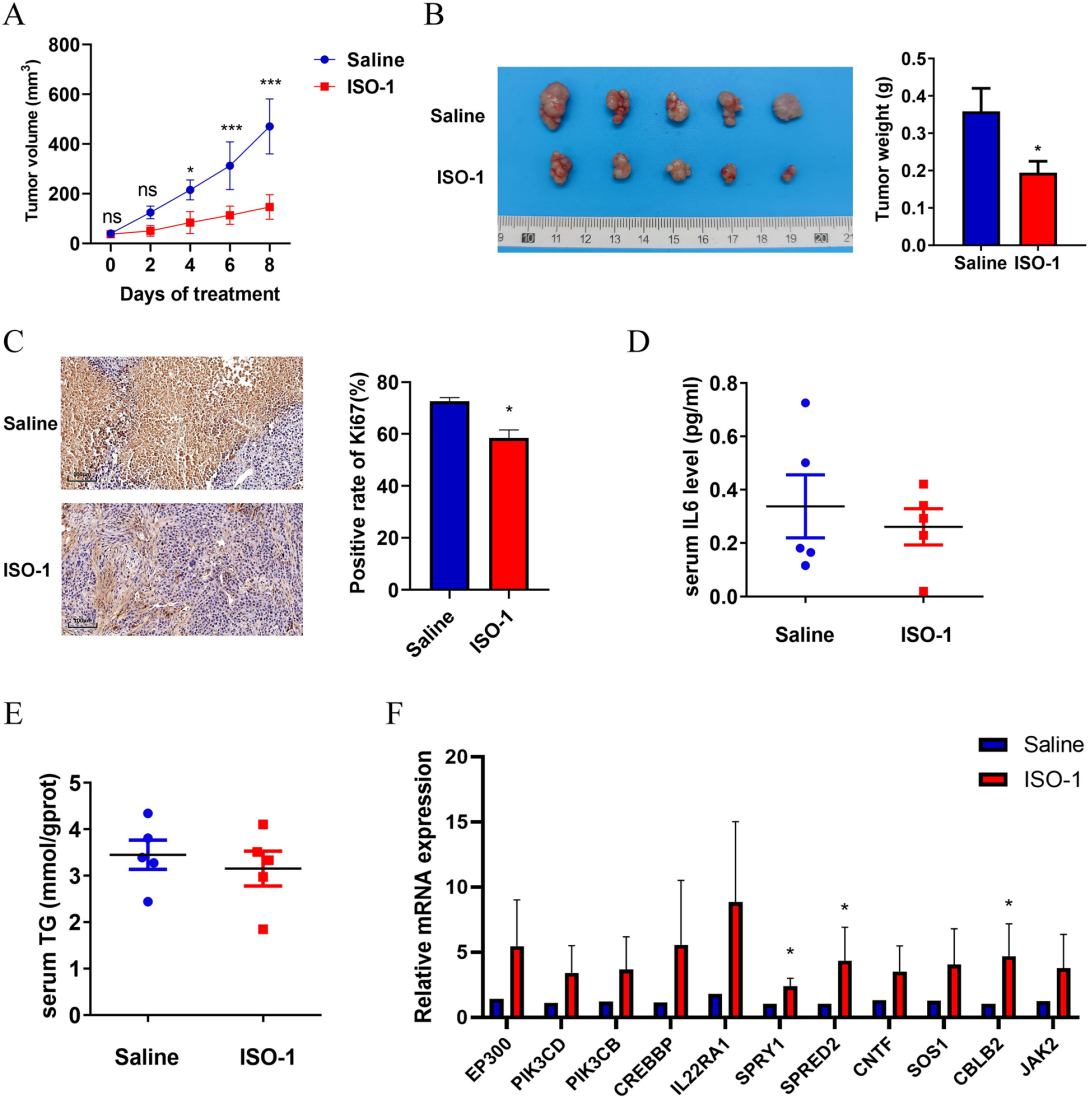
MIF antagonist ISO-1 inhibits the tumorigenicity of Hep2 cells in vivo. Four-week-old nude mice (five mice per group) were subcutaneously injected with the Hep2 cells (4 * 10.^6^ cells each mouse) and treated with ISO-1 at day eight. **A** The tumour growth curves in vivo. **B** Picture of isolated tumors and tumour weight. **C** Images and statistic results of a positive rate of Ki67. **D** Serum IL-6 level and (**E**) serum TG level were assessed by ELISA assay. **F** qRT-PCR was applied to detect relative mRNA expression of JAK/STAT gene sets. The data are presented as mean ± SD. *P < 0.05; ***P < 0.001

To evaluate whether MIF plays a role in tumorigenicity in vivo, ISO-1, a MIF antagonist, was administered to tumour-bearing mice. ISO-1 significantly inhibited tumour growth in vivo (Fig. 5A). Tumour weight (Fig. 5B) was markedly decreased in mice injected with ISO-1 compared with control mice. Furthermore, the positive rate of Ki67, which reflects the proliferation of cells, was markedly lower in the ISO-1 treatment group (Fig. 5C). In contrast with the results of the cell experiment, the concentrations of serum IL-6 (Fig. 5D) and serum TG (Fig. 5E) were decreased with ISO-1 treatment. To explore the mechanism by which cancer cells adapt to the hypoxic microenvironment, an RT-qPCR assay was conducted to test the mRNA expression of JAK/STAT pathway genes in tumour xenograft tissues. The results were basically consistence with the cellular data (Fig. 5F). In summary, all these results suggest that hypoxia activates the IL-6/JAK/STAT pathway via MIF.

## Discussion

Lipids, nucleic acids and proteins are important components of biological membranes and the structure of cells. In addition, lipid droplets are the main sites for energy storage. Lipids are involved in metabolism and play important roles in the molecular signaling of a variety of cellular activities. The regulation of lipid metabolism, such as lipid uptake, hydrolysis and synthesis, is important for maintaining cell homeostasis. In the process of tumour progression, tumour cells alter lipid metabolism to maintain survival, rapid proliferation, metastasis and chemoresistance due to changes in nutrients in the tumour microenvironment.

Under hypoxic conditions, malignant cells increase their utilization of extracellular lipids to meet the demand for bioenergy and biosynthesis and to maintain membrane homeostasis. FA metabolism supports tumorigenesis, progression and treatment resistance by enhancing lipid synthesis, storage and usage. In addition, tumour cells have strong capacity in adjusting FA metabolism and respond to extraneous and systemic metabolic signals (such as tumour therapy and obesity) to promote aggressiveness, treatment resistance and the development of related diseases [8]. In the present study, hypoxia increased TG and NEFA levels in Hep2 cells, and then we screened FA metabolism-related genes in hypoxic gene sets of laryngeal cancer. MIF, ENO2 and LDHA were identified to be related to lipid metabolism when laryngeal cancer cells were exposed to hypoxia, which is consistent with the RNA-seq analysis of Hep2 cells exposed to hypoxia. MIF is overexpressed and secreted in multiple cancer cells, particularly in response to hypoxia. As a multifunctional inflammatory cytokine, MIF is related to tumorigenesis, angiogenesis and metastasis of various malignant phenotypes. MIF can potentially promote tumorigenesis by inhibiting the classic tumour suppressor gene p53 and by regulating apoptosis in response to DNA damage and cell cycle arrest [9]. In addition, inhibition of MIF can reduce the formation of the premetastatic microenvironment in the liver and the metastasis of cancer cells [10].

In recent years, MIF was reported to be associated with lipid metabolism in multiple diseases. Changes in the expression levels of MIF have an important impact on metabolism and the immune regulation of adipose cells. MIF deficiency aggravates hepatic lipid accumulation when mice were exposed to an energy-rich fructose diet [11]. MIF upregulation reduced lipolysis and increased lipogenic pathways in adipose tissue [12]. The lack of MIF reduced chronic inflammation in white adipose tissue [13]. In this study, increased TG and NEFA levels were induced by hypoxia via the upregulation of MIF by the antagonist ISO-1. ISO-1 reversed the expression pattern of lipid metabolism-related genes, such as PPARA, PPARG, SREBF1, FASN, and LPIN2. Through a subcutaneous xenograft model, tumour growth was significantly restricted with ISO-1 treatment. Consistently, the serum IL-6 and TG levels of mice showed the same trend. However, there were no statistically significant differences, possibly due to large individual variations. A study using more mice is needed for a reliable conclusion.

In addition to the ability of MIF to counteracting glucocorticoid-induced anti-inflammatory responses, and its key role in the inflammatory cascade, MIF also participates in regulating the malignant phenotypes of various cancers by activating multiple signaling pathways. MIF expression is abnormally increased and supports the proliferation, migration and invasion of gastric cancer, breast cancer, lung cancer and pancreatic cancer cells [14–17]. MIF acted as an autocrine growth factor that promoted pancreatic cancer cell proliferation [18]. In bladder cancer cells, activation of CXCL2/MIF-CXCR2 signaling aggravated MDSC accumulation and expansion in the bladder cancer TME [19]. The MIF/CXCR7/AKT pathway drives growth and metastasis in castration-resistant prostate cancer cells [20].

To further analyse the potential mechanism of MIF in regulating lipid metabolism, we screened the related pathways through GSEA and found that the top pathway was the JAK/STAT pathway. Through the intersection of JAK/STAT pathway gene sets and RNA-seq, 14 genes were shown to have the same expression patterns, and the RT–qPCR results basically verified these patterns in vitro and in vivo. Due to large individual variations in each mouse, some genes did not show a significant difference even though the trend of gene expression of xenograft tumour tissues was consistent with the cellular results. MIF is an upstream modulator of IL-6, and IL-6 is the classical upstream activator of the JAK/STAT pathway. IL-6 binds to its receptor IL-6R and induces homodimerization and the formation of a high affinity receptor complex. This activates the MAPK and PI3K/AKT pathways to regulate the growth, differentiation, survival and chemoresistance of cancer cells [21–23].

The JAK/STAT signaling pathway is continuously activated and overexpressed in various tumour cells. The continuously activated JAK/STAT pathway in the tumour microenvironment can inhibit the antitumor immune response of immune cells, and IL-6/JAK/STAT signaling has become a popular signal target for tumour therapy [24]. MIF promotes the secretion of the inflammatory factor IL-6 in the hypoxic tumour microenvironment. It also promotes the progression of laryngeal cancer and induces changes in lipid metabolism. Thus, the MIF/IL-6/JAK/STAT pathway is expected to provide a new approach for the treatment of laryngeal cancer.

### Comparisons with other studies and what the current work adds to existing knowledge

Previous studies revealed that MIF was associated with lipid metabolism in multiple diseases. In addition to its function in lipid metabolism, the present study showed that MIF could improve can improve laryngocarcinoma cells’ adaptability to the hypoxic microenvironment by changing ways of lipid metabolism, which is a potential target for the treatment of laryngocarcinoma.

### Study strengths and limitations

There are several strengths to this study. First, the present study used both bioinformatics analysis and RNA-seq to explore the molecular mechanism involved in hypoxia-induced dysregulation of lipid metabolism in laryngocarcinoma. Moreover, this study showed that targeting lipid metabolism such as MIF might be a promising therapeutic option for the treatment of laryngeal cancer. However, the present study still remains some limitations. The first limitation is that the specific regulation of mechanism of MIF/IL-6/JAK-STAT pathway really needs more evidences to support. Another limitation is that oxygen content in solid tumour tissue was not investigated, more related studies needed to be taken in the future investigation.

## Conclusion

Hypoxia induced reprogramming of lipid metabolism in Hep2 cells through MIF/IL-6/JAK-STAT pathway. This study revealed one mechanism that allows laryngocarcinoma cells adapt to hypoxic tumour microenvironment. Therefore, a drug targeting MIF/IL-6/JAK-STAT pathway might be a promising therapeutic option for the treatment of laryngocarcinoma.

## Supplementary Information

**Additional file 1. **Primer sequences for qPCR.

## Data Availability

The following information was supplied regarding data availability: The gene expression profiles containing the clinical follow-up information is available at TCGA website(https://www.cancer.gov/about-nci/organization/ccg/research/structural-genomics/tcga). Primer sequences for PCR are attached in Supplementary Data. The other data used to support the findings of this study are available from the corresponding author upon request.

## Abbreviations

HNSCC: Head and neck squamous cell carcinoma
ELISA: Enzyme-linked immunosorbent assay
GSEA: Gene set enrichment analysis
IHC: Immunohistochemistry
NEFA: Non-esterified free fatty acids
FA: Fatty acid
TG: Triglycerides
HIF1A: Hypoxia inducible factor 1 subunit alpha
MIF: Macrophage migration inhibitory factor
LDHA: Lactate dehydrogenase A
ENO2: Enolase 2
